# A simulation model to quantify the efficacy of dry cleaning interventions on a contaminated milk powder line

**DOI:** 10.1128/aem.02086-24

**Published:** 2025-04-17

**Authors:** Devin Daeschel, Long Chen, Claire Zoellner, Abigail B. Snyder

**Affiliations:** 1 Department of Food Science, Cornell University Department of Food Science 171522, Ithaca, New York, USA; 2 College of Mechanical and Electronic Engineering, Northwest A&F University 12469, Yangling, Shaanxi, China; 3 iFood Decision Sciences, Inc., Seattle, Washington, USA; Anses, Maisons-Alfort Laboratory for Food Safety, Maisons-Alfort, France

**Keywords:** food-borne pathogens, dry sanitation, dry cleaning, flushing, Monte Carlo simulation, computer modeling, low moisture food, milk powder

## Abstract

Outbreaks of *Salmonella* in low moisture foods have been caused by cross-contamination from the processing environment into product. We used Monte Carlo simulations to model the impact of hypothetical cross-contamination scenarios of *Salmonella* from production equipment into milk powder. Model outputs included the quantity and extent of the contaminated product from a production line. Outputs were used to compare the efficacy of cleaning interventions. Cross-contamination of potential dry cleaning surrogates was also modeled. Input parameters for the model included log reductions from wiping an inoculated surface with a dry towel and transfer coefficients from an inoculated surface to milk powder. After a 2-log CFU contamination breach (*Salmonella* introduced to an 8.4 cm^2^ stainless-steel surface on the processing line before production), the number of consumer-sized milk powder units (300 g) contaminated with *Salmonella* was 72 [24, 96] (median [p5, p95] across 1,000 simulation iterations). The average concentration of *Salmonella* within contaminated units was −2.33-log CFU/g [–2.46, –1.86]. Wiping the contaminated surface with a dry towel before the production of milk powder reduced the number of contaminated units to 26 [12, 64]. Flushing the contaminated surface with 150 kg of milk powder prior to milk powder production reduced the number of contaminated units to 0 [0, 41]. Flushing with 300 kg of milk powder further reduced the number of contaminated milk powder units to 0 [0, 16]. *Enterococcus faecium* resulted in a similar number of contaminated units (74 [44, 93]) compared with *Salmonella* (72 [24, 96]) after a 2-log CFU contamination breach.

**IMPORTANCE:**

This work demonstrates the utility of modeling as a decision support tool to (i) estimate Salmonella cross-contamination into product under different scenarios, (ii) compare different cleaning interventions, and (iii) help inform the selection of a Salmonella surrogate for cleaning validation studies. Risk models can describe the tradeoffs associated with different dry cleaning strategies in low moisture food environments. For example, the model presented in this study can estimate the differences in product contamination as a consequence of flushing a processing line with increasing quantities of material. Additionally, outputs from this model can be used to evaluate the risk of cross-contamination from a contaminated dry cleaning tool. Finally, comparing outputs from a simulation model is an alternative method for comparing Salmonella surrogates used in dry cleaning validation. Simulation model outputs (i.e., prevalence and concentration of contaminated units) may be more broadly interpretable than comparing transfer coefficients alone, enhancing decision support.

## INTRODUCTION

Recent outbreaks linked to powdered infant formula, cereals, and peanut butter (1) as well as outbreaks of *Salmonella* and recalls linked to milk powder and other dry dairy products (2–6) have underscored the challenge of environmental cleaning and sanitizing in low moisture food (LMF) facilities. In 2014, the U.S. Food and Drug Administration (FDA) conducted a sampling survey of 55 milk powder processing facilities and detected *Salmonella* in 5.5% of the facilities (7). *Salmonella* can persist in low moisture processing environments (8), cross-contaminate low moisture foods (9), and then persist in contaminated food products (10). Investigations of past outbreaks linked to dry dairy products are frequently inconclusive, but some have identified processing line equipment as the likely source of contamination (8, 11).

Dry processing operations rely on dry cleaning strategies such as brushing, vacuuming, and material flushing to maintain a hygienic processing environment without introducing water. Flushing (also referred to as product push-through, dry purging, or dry rinsing) is a sanitation strategy in which food or another dry material is run through the processing line. This removes food, allergenic residues, or, in some cases, microbial contaminants before the material is discarded (12, 13). These practices are effective at removing visible food residues and are an integral part of product changeovers (14); however, research on the efficacy of these techniques for microbial removal and reducing the risk of cross-contamination is limited (15, 16). Additionally, defined parameters for the controlled implementation of physical dry cleaning regimes have not been established. As a result, physical dry cleaning methods such as flushing have not been recognized as sanitation breaks in LMF processing by the U.S. FDA (16, 17).

On the other hand, the efficacy of aqueous chemical sanitizers for microbial reduction is well studied in bench-scale experiments (18–20), but the increased risk of microbial growth from the introduction of water to a LMF processing environment remains a significant trade-off (21). There is currently a knowledge gap around the efficacy of different dry cleaning methods in reducing the risk of pathogen cross-contamination. Computer modeling as a tool to quantify cross-contamination and estimate risk trade-offs of different sanitation regimens has previously been applied to other food processing systems such as the slicing of deli meats and flume washing of lettuce (22–28). In these models, bacterial transfer rates between surfaces and food products are typically measured experimentally across different processing conditions and then used to build probability distributions that estimate a range of possible cross-contamination outcomes (29). These models then form the basis for digital tools that have broad application in decision support by helping the industry optimize their sanitation programs and reduce the risk to public health (30, 31). However, application to LMF systems to compare the efficacy of different dry cleaning methods is currently limited (32).

The objectives of this study were to (i) build a simulation model of environmental *Salmonella* transfer into milk powder during a production run, (ii) estimate the prevalence and concentration of *Salmonella* in contaminated milk powder units produced during a production run after a contamination breach, (iii) estimate the effects of milk powder flushing and wiping with a dry towel on the prevalence and concentration of contaminated milk powder units, (iv) estimate the amount of cross-contamination from a contaminated cleaning tool, and (v) evaluate potential surrogates for *Salmonella* that can be used in future dry sanitation research.

## MATERIALS AND METHODS

### Modeling a milk powder production run

Milk powder production in a dry food processing environment was modeled in this study. Processing parameters for the production run were chosen to reflect those of a typical, industrial milk powder manufacturer. Each modeled production run produced 30,000 kg of milk powder, partitioned into 100,000 units, each containing 300 g of milk powder (consumer-sized units). Milk powder production typically involves pasteurization of liquid milk, concentration by evaporation or reverse osmosis, formation of powder via spray drying, and then transportation and packaging of the final product (33). The transportation step between powder formation and packaging where milk powder flows through a filler pipe was chosen as the site of the contamination breach and source of subsequent cross-contamination. The main outputs from the model are the number of milk powder units that were contaminated during the production run (prevalence) and the mean concentration of contamination (log CFU/g) within the contaminated units. All modeling and statistical analyses were done in R version 4.2.2 (34). Each model was simulated with 1,000 iterations, with each iteration representing a single production run. The results for each simulated scenario are reported as the median [5th, 95th percentile] of the model outputs of 1,000 iterations. Preliminary testing showed that increasing the number of iterations did not meaningfully change the model outputs.

**Fig 1:**
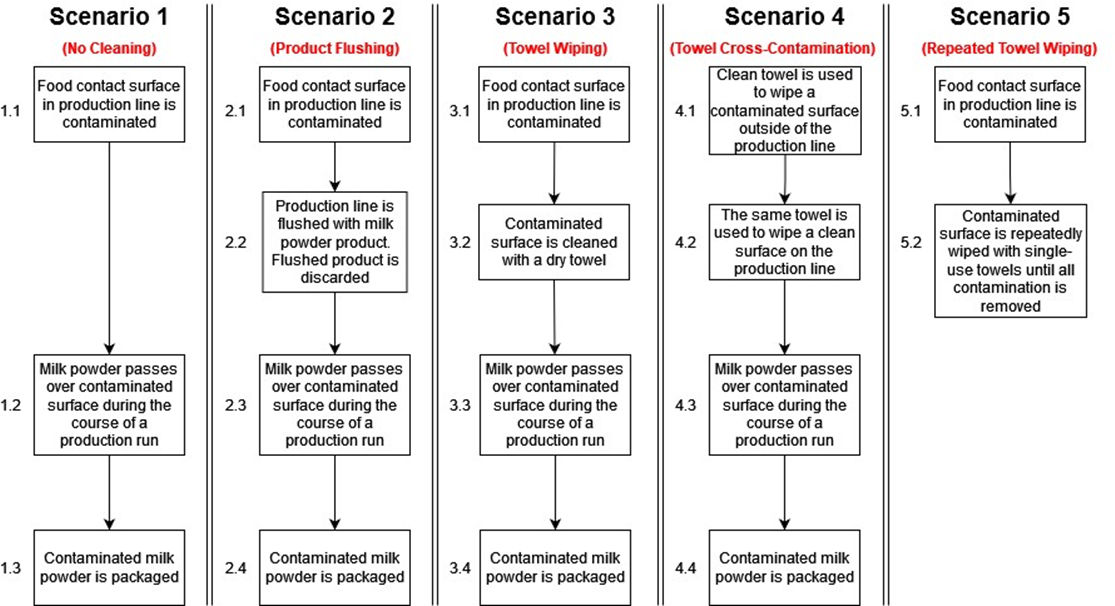
The different scenarios considered in the model. Scenario 1 (cross-contamination from a contaminated surface into milk powder), scenario 2 (cross-contamination from a contaminated surface into milk powder after a flushing intervention), scenario 3 (cross-contamination from a contaminated surface into milk powder after a dry towel wiping intervention), scenario 4 (cross-contamination from a contaminated towel to a stainless-steel surface, and then into milk powder), and scenario 5 (repeated towel wiping of a contaminated stainless-steel surface without production of milk powder).

Five different *Salmonella* cross-contamination and intervention scenarios were modeled (Fig. 1). In scenario 1, a stainless-steel food contact surface (2.4 cm in width, 3.5 cm in length, 8.4 cm^2^) within a filler pipe on the production line was contaminated with *Salmonella* prior to a production run. Three hypothetical initial surface contamination levels (2-log CFU, 4-log CFU, and 6-log CFU per 8.4 cm^2^) were tested. Scenarios 2 and 3 incorporated different dry cleaning strategies prior to the start of production (flushing and wiping with a dry towel, respectively). Scenario 4 modeled the introduction of contamination from the cleaning intervention tool, a dry towel. In this scenario, a dry paper towel was used to clean a contaminated surface (6-log CFU) and then used again on the surface of the production line. Scenario 5 simulated a more rigorous dry cleaning intervention, repeated wiping of the contaminated surface with a dry towel, to estimate how many passes of the towel are needed to remove all the surface contamination prior to production. These estimates were compared across potential bacterial surrogates (*Escherichia coli*, *Enterococcus faecium*, and *Listeria innocua*) for *Salmonella* during dry cleaning validation and verification activities.

### Modeling initial contamination

Each iteration in the base model started with a user-defined quantity of initial contamination on the processing line. There is limited published research enumerating *Salmonella* from food processing surfaces in real-world or realistic scenarios, but one study reported that the *Salmonella* contamination on processing surfaces averaged 1.3 CFU/cm^2^ after a production run with contaminated dry poultry feed (35). Target aerobic plate count values for food processing surfaces may be set as hygienic indicators. These values typically vary depending on the environment being sampled, but less than 500 CFU prior to sanitization is recommended as acceptable by the Almond Board of California (36). We chose 2-log, 4-log, and 6-log CFU as starting contamination loads introduced on the contaminated surface (8.4 cm^2^) to encompass a range of hypothetical initial contamination levels. The 2-log CFU load was considered the most likely initial contamination level and was used as the primary point of comparison between scenarios.

### Modeling microbial transfer and reduction

The main function of the model simulates the transfer of bacteria from a contaminated stainless-steel surface (filler pipe) into milk powder. Transfer data for *Salmonella enterica, E. faecium, L. innocua*, and *E. coli* from a spot inoculated stainless-steel coupon into 10 g quantities of milk powder were generated in the present work (described below, data available in File S1). Probability distributions (Table 1) were fit to the transfer data using the fitdist function in the “fitdistrplus” package version 1.1.8 (37). For transfer from a spot inoculated surface to milk powder, transfer coefficients were log-transformed and then fitted to a normal distribution (29, 38). Goodness of fit was measured with the Kolmogorov–Smirnov test and Q-Q probability plots. Simulated production runs were modeled through the following steps for each iteration:

Select the starting contamination (*C*_0_) on the stainless-steel filler pipe:


$$C_{0}=(2\;log,4\;log,or\;6\;log\;CFU)$$

**TABLE 1:** Model input parameters, distributions, and references for microbial transfer and reduction during milk powder processing

Model parameter (symbol)	Distribution	Flow chart steps	Organism	Distribution inputs		Input units	Data source
Transfer coefficient from spot-inoculated stainless-steel surface to milk powder (T_c_)	Log-normal	1.2, 2.3, 3.2, 3.3, 4.3		μ	σ	Log transfer coefficient	Current study
			*S. enterica*	−2.29	0.515		
			*E. coli*	−1.23	0.388		
			*L. innocua*	−1.68	0.412		
			*E. faecium*	−2.34	0.254		
Microbial reduction on spot-inoculated stainless steel surface from wiping with a dry towel (*M_r_*)	Uniform	3.2, 5.2		Min	Max	Log reduction	(39)
			*S. enterica*	0.069	0.892		
			*E. coli*	0	0.421		
			*L. innocua*	0.021	0.716		
			*E. faecium*	0.439	1.95		
Transfer coefficient from inoculated dry towel to stainless steel surface (*T_d_*)	Log-normal	4.2		μ	σ	Log transfer coefficient	Current study
			*S. enterica*	−4.32	0.615		
			*E. coli*	-^*a*^	-		
			*L. innocua*	-	-		
			*E. faecium*	-	-		
Proportion of milk powder that contacts contaminated surface before packaging (*P_mp_*)	Uniform	1.3, 2.4, 3.4, 4.4		Min	Max	Percentage	Assumed
			*S. enterica*	3%	20%		
			*E. coli*	3%	20%		
			*L. innocua*	3%	20%		
			*E. faecium*	3%	20%		
CFU transferred from stainless-steel surface to dry towel (N_CFU_)	Uniform	4.1		Min	Max	CFU	(39)
			*S. enterica*	20	5600		
			*E. coli*	-	-		
			*L. innocua*	-	-		
			*E. faecium*	-	-		

^*a*^ -, these data were not collected for the given organism.

Sample a transfer coefficient (*T_c_*) from the surface to milk powder log-normal transfer coefficient distribution:


$${Log(T}_{c})∼\text{LogNormal}\left(\mu ,\sigma \right)$$

Calculate the number of CFU transferred (*N_t_*) into each 10 g quantity of milk powder that contacts the contaminated surface at contact event *t*, where *t* is each contact event between a 10 g quantity of milk powder and the contaminated surface.


$$N_{t}∼\text{Binomial}\left(C_{t-1},T_{c}\right)$$

Update the remaining contamination on the surface (*C_t_*) to reflect the CFU that was transferred in the previous contact event:


$$C_{t}=C_{t-1}-N_{t}$$

Repeat Steps 3 and 4 until all milk powder (30,000 kg) is run through the production line or until all contamination on the surface has been transferred (*C_t_* = 0).

It is unlikely that the entire quantity of milk powder moving through a processing line would contact a single contaminated surface site. Only a fraction of milk powder moving through the line would have the chance to contact the contaminated surface in the filler pipe and for microbial transfer from the surface to occur. This same principle would apply to a niche or “dead zone” on the processing line, which the milk powder only infrequently contacts. Therefore, we implemented a parameter in the model to account for the proportion of milk powder in a single product unit (300 g) that contacts the contaminated surface before being packaged. We assumed that anywhere from 3% (10 g) to 20% (60 g) of the milk powder entering a product unit would contact the contaminated surface. Since microbial transfer was calculated for each 10 g of milk powder passing through the line, a single milk powder product unit (300 g) could contain a minimum of 10 g and a maximum of 60 g of milk powder exposed to contamination, representing 1–6 total transfer events for all milk powder in that unit, respectively. At the end of an iteration, the proportion of milk powder (*P_mp_*) in each unit that contacted the contaminated surface is sampled from a uniform distribution:


$$P_{mp\;}\;∼\;Uniform\left(3\%,20\%\right)$$

The sampled proportion was then used to calculate the number of CFU in each milk powder unit. This stochasticity was modeled with a uniform probability distribution, which is typical in situations where there are no *a priori* data (40).

A fourth parameter was added to model scenario 3 (wiping with a dry towel intervention). This parameter represented the microbial reduction (*M_r_*) when a dry towel is used to wipe the contaminated surface. This was described using a uniform distribution with minimum and maximum values taken from microbial reduction data (Table 1):


$$M_{r\;}\;∼\;Uniform\left(min,\;max\right)$$

Microbial reduction data for *Salmonella* on a spot inoculated stainless-steel coupon wiped with a dry towel and data on the microbial transfer to the towel were previously reported by Chen et al. (39) (File S1).

For scenario 4, an additional parameter (*T_d_*) for the transfer from a contaminated dry towel to a clean surface was included using a transfer coefficient log-normal probability distribution (Table 1):


$${Log(T}_{d})∼\text{LogNormal}\left(\mu ,\sigma \right)$$

Transfer data for *S. enterica* on an inoculated dry towel to a clean stainless-steel surface was generated in the current study (File S1). In scenario 4, the dry towel initially became contaminated from being used to wipe a contaminated surface outside of the modeled processing line. The amount of contamination on this initial surface was set to 6-log CFU to represent a worst-case scenario. The amount that gets transferred to the dry towel from the surface was modeled using a uniform distribution:


$$N_{CFU}∼\text{Uniform}\left(min,\;max\right)$$

Minimum and maximum amounts of CFU transferred from the surface to the towel were reported by Chen et al. (39).

### Sensitivity analysis

Starting contamination (*C_0_*), the transfer coefficient from a stainless-steel surface to milk powder (*T_c_*), microbial reduction during dry cleaning (*M_r_*), and the proportion of milk powder that contacted the contaminated surface within each unit (*P_mp_*) were included as parameters in a global sensitivity analysis to determine their relative effects on the prevalence and concentration of contaminated milk powder units during a scenario 3 (towel wiping) production run. Scenario 3 was chosen for the sensitivity analysis so that microbial reduction from wiping with a dry towel could be included as a parameter. Sensitivity analysis was performed using partial rank correlation coefficients (PRCC) and was conducted with the “epiR” package version 2.0.66 in R (41). The model makes several necessary simplifications that may affect how representative the model is of real-world scenarios. For example, we assume that the rate of transfer is independent of the starting contamination and that bacterial transfer events are independent of each other, which is typical of similar models of microbial transfer (29, 38). However, it should be noted that in microbial transfer experiments, a higher initial bacterial population tends to correspond to lower transfer coefficients (39, 42, 43), but this correlation can change, depending on the organism (44). We also did not implement the possibility of contamination being spread or re-introduced in the processing line from cross-contaminated milk powder. In other words, *Salmonella* could not transfer to a new surface from the milk powder itself. For a given production run, we also assumed that the sampled transfer coefficient and proportion of milk powder in each unit that contacted the contamination were constant for the duration of that simulation. Finally, the intervention using dry towels was done with three passes of the towel, each with a standardized sheer stress which, while experimentally consistent, may not be accurate to real-life wiping done by a human.

### Bacterial strains

The same bacterial strains were used in our previous study associated with dry sanitation (39). The *Salmonella* strain chosen for this study was *Salmonella* Enteritidis PT30, since it has been associated with an outbreak and has previously been used in low moisture food research. The surrogate strains used in this study were *E. faecium* NRRL B-2354, *L. innocua* ATCC 51742, and *E. coli* ATCC 25922. *E. faecium* NRRL B-2354 was chosen because it has been used as a thermal processing surrogate for *Salmonella* (45). The *L. innocua* and *E. coli* strains were chosen, since both have been used in prior surface attachment studies (46, 47).

### Inoculum preparation

The same inoculum preparation method was used as described by Chen and Snyder (39) .Briefly, a loopful of frozen stock for each bacterium was inoculated into Brain Heart Infusion (BHI) broth (BD, Thermo Fisher Scientific, Waltham, MA, USA) and incubated at 37°C for 24 h. Overnight broth suspensions were streaked onto BHI agar plates and incubated at 37°C for 24 h. An isolated colony was transferred from stock plates into BHI broth, followed by incubation at 37°C for 24 h. After incubation, the culture was centrifuged (Eppendorf 5804R, Eppendorf, NY, USA) at 10,000 revolutions per minute (RPM) for 5 min, and the cell pellet was washed twice in 0.1% phosphate-buffered saline (PBS) (BD, Thermo Fisher Scientific, Waltham, MA, USA). After washing, the cell pellets were resuspended in the same volume of 0.1% PBS to achieve a cell concentration of ~9.0 log CFU/mL. Inoculation procedures were repeated for each of the four bacteria using three biological replicates.

### Surface inoculation and microbial transfer

Microbial reduction of an inoculated stainless-steel surface wiped with a dry towel, and transfers from the inoculated surface to the towel were measured previously by Chen et al. in triplicate (39). Transfer coefficients from an inoculated stainless-steel surface to milk powder and for an inoculated dry towel to a stainless-steel surface were generated as follows:

#### Stainless-steel surface to milk powder

We opted for a spot inoculation method to represent *Salmonella* introduced during a wet cleaning or moisture breach event (i.e., leak or flood) and then left to dry on the surface; however, this would not be typical of different contamination events such as *Salmonella* attaching to a surface from incoming ingredients (39). Eighteen 10 µL drops of inoculum were spot inoculated on sanitized stainless-steel coupons (2.4 cm in width, 3.5 cm in length, and 0.48 cm in thickness, AISI 304 stainless-steel 2B finish). Then, the coupons were dried in a biosafety cabinet overnight with the fan on (Thermo Fisher Scientific, Waltham, MA, USA). Each inoculated coupon was manually shaken and mixed with 10 g of milk powder (Nestle Carnation, Switzerland) in a sterile sample bag for 5 min to facilitate the microbial transfer from the inoculated coupon to milk powder. The cells were collected and enumerated from: (i) coupons before the transfer treatment to milk powder, (ii) coupons following the transfer treatment, and (iii) the milk powder following the transfer treatment. Briefly, coupons were rinsed with 10 mL 0.1% PBS in sample bags (Whirl-Pak, Madison, WI, USA), and 10 g milk powder was diluted with 20 mL 0.1% PBS in sample bags. The samples were diluted and spread plated on Brain Heart Infusion (BHI) (BD, Thermo Fisher Scientific, Waltham, MA, USA) and then enumerated. Microbial transfer experiments were conducted for five replicates for each bacterium.

#### Dry towel to stainless-steel surface

A dry towel was inoculated as follows: glass beads were first inoculated with each of four bacterial strains according to the same method described by Chen et al. (39). Paper towels were autoclaved to remove any preexisting microbial contaminants (48) and then inoculated through manual mixing and rolling with glass beads (Walter Stern Inc., Port Washington, NY, USA) for 1 min. Stainless-steel coupons were first sanitized in 10% bleach for 5 min, then wiped with dry towels, soaked in 70% ethanol for 2 min, wiped with a dry towel, and then air-dried overnight before treatment. Microbial transfer from an inoculated dry towel to stainless-steel coupons was done through a simulated dry cleaning process described by Chen et al. (39). The cells were collected from unused dry towels, coupons following the cleaning treatment, and the dry towel following the cleaning treatment and enumerated with serial dilution and spread plating on Brain Heart Infusion (BHI) (BD, Thermo Fisher Scientific, Waltham, MA, USA). Briefly, dry towels were mixed with 10 mL 0.1% PBS in sample bags (Whirl-Pak, Madison, WI, USA) and coupons were diluted with 2 mL 0.1% PBS (BD, Thermo Fisher Scientific, Waltham, MA, USA) in sample bags which yielded a detection limit of 0.30-log CFU. Microbial transfer experiments were conducted for five replicates for each bacterium.

## RESULTS AND DISCUSSION

### The parameters for transfer coefficient and the proportion of milk powder that contacted the contaminated surface were highly correlated with product contamination outcomes

**Fig 2:**
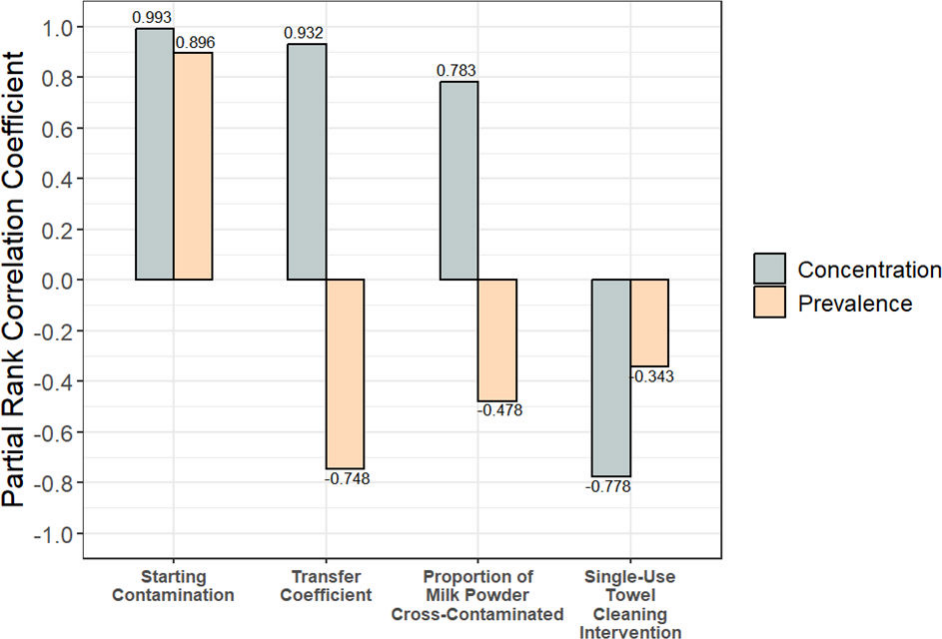
Partial rank correlation coefficients for all model input parameters were correlated with the prevalence and concentration of contaminated units (scenario 3). The transfer coefficient was highly influential on concentration and prevalence.

Through a global sensitivity analysis of scenario 3, it was found that the parameters most correlated with both the prevalence (number of contaminated units) and concentration (log CFU/g within units) of *Salmonella* were the starting contamination level (*C_0_*), the transfer coefficient from stainless steel to milk powder (*T_c_*), and the proportion (*P_mp_*) of milk powder in each product unit that contacted the contaminated surface (Fig. 2). Both the transfer coefficient and the proportion of milk powder contacting the contamination were positively correlated with concentration but negatively correlated with prevalence. This is because a higher transfer coefficient and a higher proportion of milk powder contacting the contaminated surface resulted in a high level of *Salmonella* transferring into a smaller number of product units. Indeed, the transfer coefficient had almost as much impact on outcomes as the amount of starting contamination (2-log, 4-log, or 6-log CFU) (Fig. 2). The proportion of milk powder in a unit that contacted the contaminated surface and the dry wiping intervention were similarly impactful on outcomes; however, dry wiping was negatively correlated (Fig. 2).

The large influence of the transfer coefficient on the prevalence and concentration of contamination underscores the importance of the primary data used for this parameter. The rates of cross-contamination are typically influential model parameters (49). Previous studies have shown that various factors can affect microbial transfer: inoculation method (39), contact time between the contaminated surface and food product (50), surface material (51, 52), and pathogen strain selection (53). Larger bacterial starting populations used in transfer experiments also tend to result in smaller transfer coefficients (39, 42, 43). Our transfer experiments used a ~ 9 log CFU/mL inoculum, which resulted in starting populations of ~4-log to 6-log CFU per coupon. Therefore, our model may overestimate the amount of transfer in low-level contamination scenarios (2-log-CFU starting contamination). Moreover, the shear stress from food and other dry materials on contaminated surfaces varies in different parts of the processing line and impacts the transfer coefficient. Future experimental research on pathogen transfer should elucidate how the flushing material, the rate of material flow, and the geometry of the processing line affect the shear stress on contaminated surfaces, and how this impacts the transfer coefficient of microbial pathogens. Additionally, the effect of the initial bacterial population size on the rate of transfer should be considered, especially for contamination scenarios with a low level of initial contamination. This will be essential to generate accurate and representative transfer coefficients for modeling applications. Transfer rates should also be accurate to the medium of interest. In this case, we modeled transfer into milk powder, but for other matrices like salt (52) or bulk almonds (54, transfer may be very different.

Similarly, the proportion of milk powder that contacted the contaminated surface was highly influential on model outputs. Our current model considers only a simplified cross-contamination event between a smooth stainless-steel surface and milk powder that passes over the surface on its way to packaging. However, contamination may be harbored in parts of the line that contact milk powder more (e.g., larger contaminated surface area) or less (e.g., niches) than what we considered in our model. For example, contamination spread out over a large surface could give more opportunities for contact with food material. Such a scenario could occur if an incoming ingredient on a line is contaminated. One study found *Salmonella* contamination in all tested areas of a spray drier after inoculated soy protein isolate was run through it (55). On the other hand, contamination isolated in a singular niche could have much less contact with food material on the line. For example, driers and conveyance equipment have niches where food material can accumulate and potentially reduce the frequency of contact between contamination harbored within the niche and successive food material on the line. More simply, any physical cracks in the processing line could reduce contact with food material, resulting in a slower diffusion of contamination during processing and reduced effectiveness of flushing. Collaboration with industry stakeholders to determine how food material interacts with problematic niches or “dead zones” within active processing lines will be critical for generating accurate parameters in more complex models.

### A contamination breach in a milk powder processing line resulted in a small number of contaminated units and a low concentration of *Salmonella* within those units

**Fig 3:**
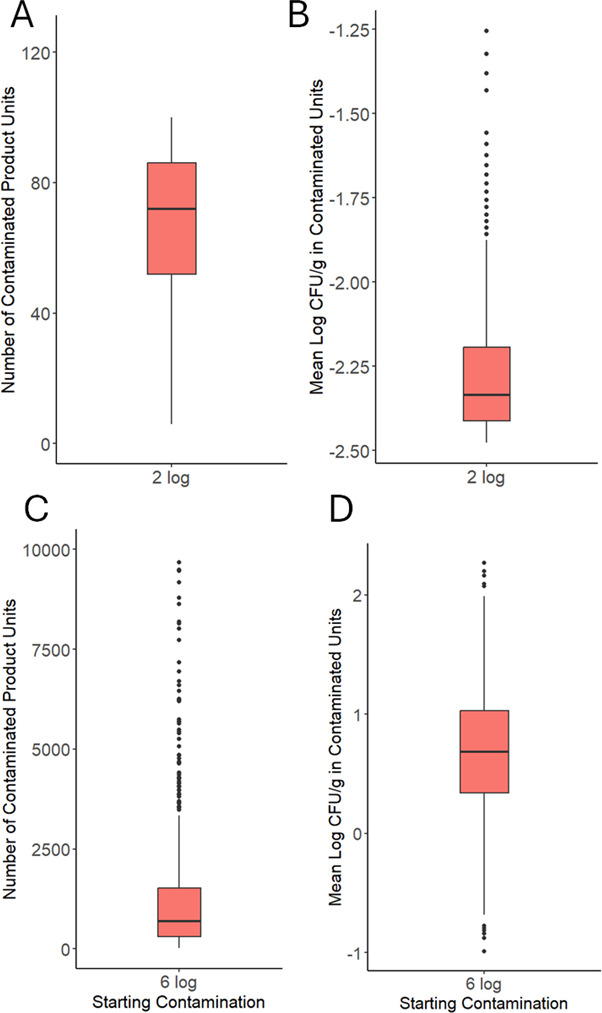
The results from 1,000 simulated production runs (scenario 1: no cleaning) are plotted for 2-log CFU (A and B) and 6-log CFU (C and D) starting contamination levels. (A and C) Each data point represents the number of milk powder units (300 g) contaminated with *Salmonella* in a simulated production run. (B and D) Each data point represents the average concentration of *Salmonella* within the contaminated milk powder units of a simulated production run.

After a 2-log CFU contamination breach in scenario 1 (Fig. 1), the number of contaminated units across simulations was 72 [24, 96] (Fig. 3A). The average concentration of *Salmonella* within contaminated units was −2.33-log CFU/g [-2.46,–1.86] (Fig. 3B). When the starting contamination level was increased to 6-log CFU *Salmonella*, the number of contaminated units increased to 688 [95, 4420], a 9.6-fold increase compared with the 2-log CFU initial contamination level (Fig. 3C). Similarly, the average concentration of *Salmonella* within contaminated units for a simulated production run increased to 0.689-log CFU/g [−0.122, 1.55] (3.2 log-increase) (Fig. 3D). Although both the number of contaminated units and the average concentration of *Salmonella* within contaminated units increased with higher initial contamination levels, the concentration increased more than the prevalence. This was also apparent in the sensitivity analysis that showed a greater PRCC between starting contamination level and concentration in the finished product than between starting contamination level and prevalence of contaminated products (Fig. 2). Notably, the variability in the number of contaminated products in each simulated production run also increased between the 2-log (standard deviation: 23 product units) and 6-log (standard deviation: 2,523 product units) contamination scenarios. Similarly, the standard deviation in average concentration also increased from 0.20-log to 0.52-log CFU/g. This is because as the amount of contamination increases so does the possible number of contaminated units and the concentration of contamination within those units.

Published data on the number of contaminated product units within recalled lots are limited; however, there are studies that have enumerated the concentration of pathogens in recalled product units (Table 2). These studies have estimated the amount of contamination within recalled dry powder products to be between −2.82 and −1.62-log CFU/g (Table 2). This concentration range was similar to what was predicted by our model following a 2-log CFU *Salmonella* breach: −2.33-log CFU/g [–2.46,–1.86] (Fig. 3B). Notably, these studies included *Cronobacter* spp., *Salmonella* Ealing, *E. coli* O121, and *Salmonella* Typhimurium in powdered infant formula, wheat flour, and baking mix, respectively (56–59). More experimental data are necessary to validate this model, particularly regarding the predicted number of contaminated units. Although data collected from recalled products provide valuable insights, a more rigorous approach would involve conducting controlled studies in a pilot-scale or industrial setting with a surrogate for *Salmonella* dry transfer. However, introducing surrogate organisms to an actual production environment presents challenges, as it may raise concerns about potential contamination and operational safety.

**TABLE 2:** Reported pathogen concentrations in recalled dry powder products^*a*^

**Recalled product details**	**Organism**	**Reported contamination concentration**	**Converted concentration (log CFU/g or log MPN/g**)	**Reference**
Powdered infant formula produced in January 2007. A 22,000 kg batch was recalled. Product was packaged and sold as two 400 g bags.	*Cronobacter* spp.	−2.78 log CFU/g	−2.78 log CFU/g	(56)
Powdered infant formula produced in 1985. Product was packaged and sold in 25 kg bags.	*Salmonella* Ealing	1–6 CFU/450 g	−2.65 to −1.87 log CFU/g	(57)
Wheat flour produced in 2016. Product was packaged and sold in 1 kg and 10 kg bags.	*Escherichia coli* O121	0.15 to 0.43 MPN/100 g	−2.82 to −2.37 log MPN/g	(58)
Raw wheat flour used in baking mix produced in 2008.	*Salmonella* Typhimurium	0.0036 to 0.024 MPN/g	−2.44 to −1.62 log MPN/g	(59)

^*a*^ Concentrations reported by studies were converted to log CFU/g or log MPN/g for ease of comparison. The reported concentrations of pathogens in recalled products were similar to what our model predicted during a contamination breach.

The predicted prevalence and concentration of contaminated milk powder units can be used to contextualize food safety risks. Assuming a 25 g serving size for milk powder, the single most contaminated product unit across all 2-log CFU simulations had 5.5 CFU of *Salmonella* per 25 g of milk powder. A future quantitative microbial risk analysis (QMRA) is necessary to quantify the risk of illness and infection from contaminated product units based on dose-response models. As a point of comparison, a dose-response model of *Salmonella* predicted 7 CFU and 36 CFU as inflection points at which infection and illness would occur in half of the exposed population, respectively (60). Importantly, the anticipated amount of contamination in a product unit does not exactly translate to the risk of illness. Any amount of ingested *Salmonella* has the potential to cause illness, and the amount of *Salmonella* in a milk powder unit could grow to more dangerous levels before exposure if temperature abuse occurs after the milk has been rehydrated by the consumer. Furthermore, heterogeneous distributions of bacteria within contaminated product units could result in some servings having a higher pathogen concentration. One study enumerating *Cronobacter* from recalled powdered infant formula found that the average contamination concentration in the recalled formula was −2.78-log CFU/g, but samples as high as 2.75-log CFU/g were detected (56). Future modeling applications should account for heterogeneous distribution within products to better predict the range of contamination levels among servings. Future work could also investigate the use of modeling to provide novel information during a root cause analysis to understand what parameters or scenarios could lead to such sporadic contamination or heterogeneous distributions of contamination. Overall, this suggests that the average contamination concentration of *Salmonella* in recalled dry powder products like milk powder is likely low for the majority of contamination breaches (< 2 log CFU). Contamination breaches may therefore go unnoticed if they result in concentrations that fall below the limit of detection in 25 g product samples (61), are present in a fraction of all units produced within a lot, and are below the level likely to cause a detected illness. However, our model also showed that if the quantity of *Salmonella* in a breach was 6-log CFU, then the *Salmonella* concentration within contaminated units increased. Breaches of this level are less likely to occur but could arise from a severe scenario like a flood or roof leak where contamination and moisture are introduced. Future modeling work should include a QMRA to investigate how elevated contamination concentrations within products correlate to detectable levels of illness or detection through a product sampling plan.

### Flushing reduced the prevalence and concentration of *Salmonella* in contaminated units after a contamination breach

**Fig 4:**
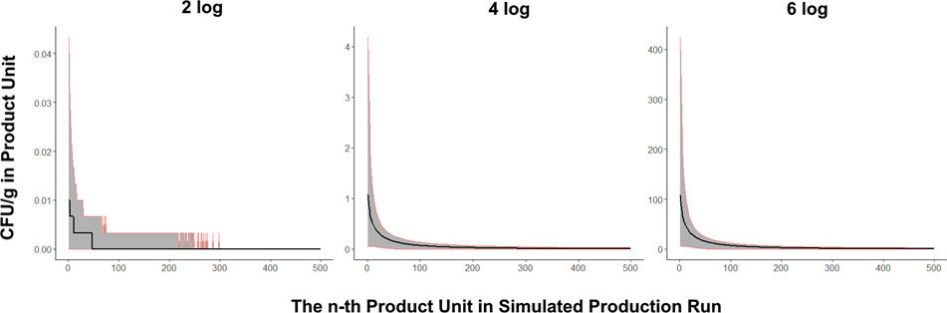
The median contamination concentration (CFU/g) in the n-th milk powder unit (i.e., from the 1st unit produced to the 500th) is graphed in black with 5th and 95th percentiles graphed in red. The concentration of contamination within product units decreased exponentially as sequential units were produced in a simulated production run (scenario 1).

Milk powder units produced directly after the contamination breach (scenario 1) had a higher concentration of *Salmonella* compared with units produced later in the production run (Fig. 4). The concentration of *Salmonella* in the first unit produced after a 2-log CFU contamination breach was 0.01 CFU/g [0, 0.04], whereas the 100^th^ unit had a contamination concentration of 0 CFU/g [0, 0.003] (Fig. 4). For a 6-log CFU contamination breach, the first and 100^th^ product units had *Salmonella* concentrations of 108 CFU/g [6.93, 424] and 6.97 CFU/g [0, 12.3], respectively (Fig. 4). This is because the bulk of the contamination transferred from the surface into the initial product units, leaving fewer cells on the surface to transfer into units later in production. This logarithmic decline in contamination across sequential product units was previously observed in a bagged lettuce model (27). In this model, transmission of *E. coli* O157:H7 into bagged lettuce was the greatest in the initial batches after contamination was introduced. Food manufacturers make use of this phenomenon by applying flushing to processing lines to remove allergenic and microbial contaminants.

**Fig 5:**
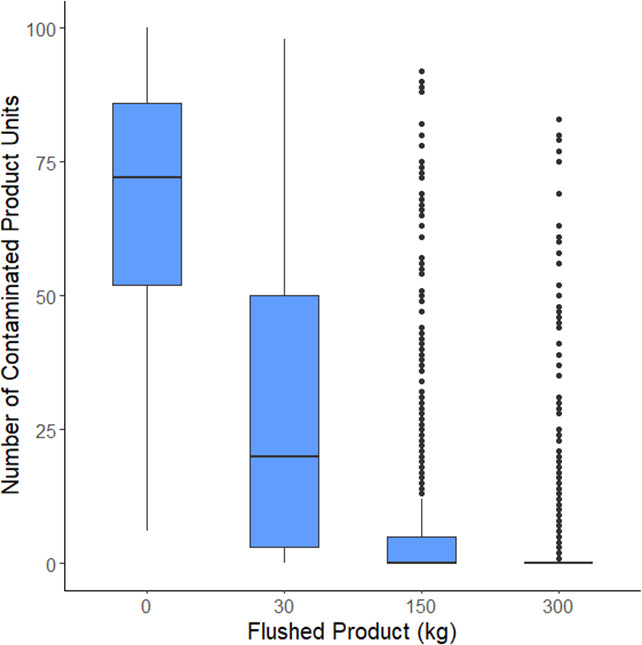
The results of 1,000 simulated production runs (scenario 2) with increasing amounts of material used in flushing before production begins (30, 150, and 300 kg). Increasing the amount of flushed material resulted in fewer product units contaminated with *Salmonella*.

To evaluate the efficacy of this intervention, we simulated flushing (scenario 2, 2-log CFU *Salmonella*) with variable amounts of milk powder (30 kg, 150 kg, and 300 kg). Increasing the amount of flushed material resulted in fewer finished products contaminated with *Salmonella* (Fig. 5). Without any flushing (scenario 1), the median number of contaminated units across all simulations was 72 [24, 96]. This was reduced to 20 [0, 82] contaminated units after flushing with 30 kg and further reduced to 0 [0, 41] units after flushing with 150 kg. Flushing with 300 kg resulted in 0 [0, 16] contaminated units. Moreover, flushing with 30 kg, 150 kg, and 300 kg resulted in 17.4%, 63%, and 79.9% of the simulated production runs having 0 contaminated units, respectively. These results suggest that flushing may quickly remove the majority of contaminating cells and reduce the amount of downstream contaminated milk powder product. In another dry flushing study, Muckey et al. (35) found that after introducing *Salmonella* into animal feed, flushing the mixers with two batches of feed decreased the concentration of *Salmonella* below the limit of detection (10 CFU) in both feed and on equipment surfaces. Liu et al. (52) tested product flushing on a bench scale set up by inoculating beads with *Salmonella* and mixing them with food material to see how much *Salmonella* was removed from the beads. Although they did find significant reductions of *Salmonella* on stainless-steel beads after flushing, they did not measure any detectable reductions on polypropylene beads regardless of how much flushing was done. This difference in microbial removal between surface materials may have been due to *Salmonella* having a stronger attachment on polypropylene surfaces. Suehr et al. (62) tested flushing in an inoculated lab-scale auger and found that *Salmonella* contamination was reduced by up to 5 logs after flushing with flour or corn meal; however, complete removal of *Salmonella* from the system was not achieved likely due to the presence of dead zones in the auger system. These results point to the strengths and limitations of flushing, but extrapolation to commercial-scale operations remains challenging. The efficacy of flushing at removing microbial contaminants will be specific to the scale of the manufacturing operation and will depend on various equipment properties, treatment parameters, as well as the material used for flushing.

The variability in model outputs between simulations should also be considered when estimating the degree of risk reduction from a flushing intervention. For example, although the 300 kg flushing intervention after a 2-log CFU breach resulted in the removal of all contamination in 79.9% of simulations, a small number of simulations still resulted in more than 50 contaminated product units (Fig. 5). This variability is driven by the surface to milk powder transfer coefficient and the amount of milk powder contacting the contaminated surface, which our sensitivity analysis showed were highly correlated with product contamination outcomes (Fig. 2). Accordingly, these parameters also influence how much *Salmonella* contamination is removed during flushing. The amount of food material used for industrial flushing procedures is often based on historical practices rather than research-backed evidence. Establishing clear parameters for flushing—such as quantity of material, material type, and pneumatic settings—is crucial to ensure the intervention is applied effectively. When developing flushing protocols, it is necessary to define the desired degree of risk reduction, while balancing food safety with economic and sustainability (e.g., loss of flushing materials, duration, and frequency of downtime) considerations specific to each processing plant’s operations.

### Wiping with a dry towel modestly reduced the prevalence and concentration of *Salmonella* in milk powder, with minimal cross-contamination to other surfaces

**Fig 6:**
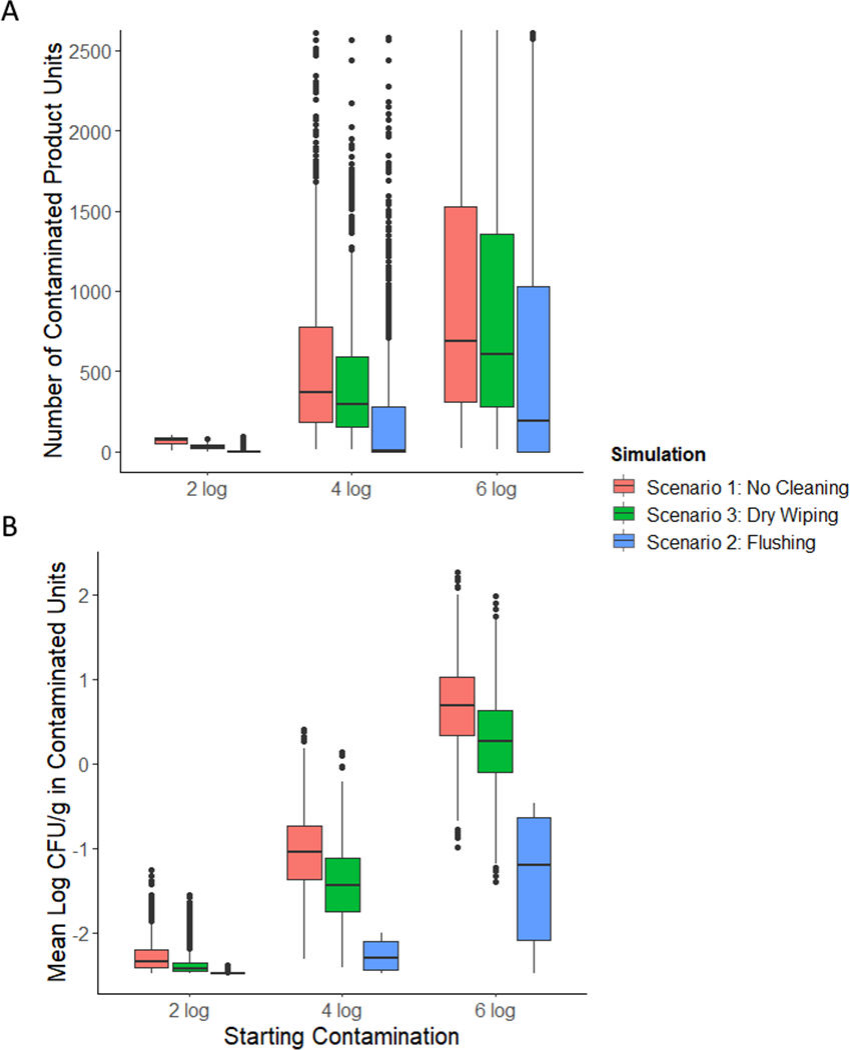
The results of 1,000 simulated production runs for scenario 1, 2, and 3 runs are plotted for each starting contamination level (2-, 4-, and 6-log CFU). (A) Each data point represents the number of milk powder units (300 g) contaminated with *Salmonella* in a simulated production run. (B) Each data point represents the average concentration of *Salmonella* contamination within the contaminated milk powder units of a simulated production run.

Simulated dry cleaning of the contaminated surface (2-log CFU) by wiping with a dry towel (scenario 3; Fig. 6) resulted in a 64% reduction in the amount of milk powder units contaminated with *Salmonella*: 72 [24, 96] to 26 [12, 64]. The average concentration of *Salmonella* within all contaminated units slightly decreased from −2.33-log CFU/g [-2.46,–1.86] to −2.42-log CFU/g [-2.48,–2.08]. In a 6-log CFU contamination breach, the percent reduction in the median number of contaminated units from a dry wiping intervention was only 11%: 688 [94, 4420] to 608 [85, 3981], but the reduction in the absolute number of contaminated units (2-log CFU breach: 46 less contaminated units, 6-log CFU breach: 80 less contaminated units) was greater. The average concentration of *Salmonella* within all contaminated units decreased from 0.69-log CFU/g [−0.12, 1.5] to 0.26-log CFU/g [−0.65, 1.8]. Mechanical dry cleaning methods such as wiping, brushing, and scraping generally offer limited removal of microbial contamination but can produce visibly clean surfaces (63) that pass ATP tests (64). Our results show how the experimentally measured log reductions from towel wiping (< 1 log CFU; Table 1) manifested as modest reductions in the number of *Salmonella*-contaminated product units. However, whether this translates to a meaningful reduction in the risk of *Salmonella* exposure must be evaluated in a QRMA.

The majority (83%) of scenario 4 simulations (cross-contamination from a dirty surface to a clean surface through towel wiping) did not result in any cross-contamination to the processing line from the towel despite a heavily contaminated initial surface (6-log per 8.4 cm^2^) that was wiped with the towel. Therefore, downstream contamination to milk powder product was also very low. The number of CFU that transferred from the original contaminated surface to the towel and then to the processing line surface was 0 [0, 2]. Of all simulations, 28 CFU was the largest number of cells ever transferred from the dry towel to the processing line. These results reflect the low transfer rates from a contaminated surface to a dry towel and then from a contaminated towel to a clean surface obtained from the experimental data (Table 1).

Cleaning tools are often cited as a potential vector for contamination in dry processing environments (12), and foodborne pathogens have previously been detected in cleaning tools (65, 66). Consequently, measures to limit cross-contamination via cleaning tools are considered an important element of good manufacturing practices (GMPs) in dry processing facilities (67, 68). Whether cross-contamination of pathogens from cleaning tools is a significant contributor to outbreaks is less clear. A panel of food safety experts rated cleaning tools as medium importance for inclusion in an environmental monitoring program for *Listeria monocytogenes*, and a plurality of experts did not consider cleaning tools to be a transfer reservoir (69); however, more data are needed to better understand the risk of contamination from cleaning tools in active manufacturing operations. Our results similarly indicate that cleaning tools may not be large drivers of cross-contamination, particularly if they are discarded or sanitized between uses, and that the relative risk of other pathogen reservoirs in the processing facility may be of more importance in protecting public health. Nonetheless, cleaning tools should still be managed as a part of GMPs (14) to ensure a hygienic processing environment. Moreover, research has shown that towels can be a vector for the transmission of allergens (70) as well as microorganisms (71, underscoring the importance of hygienic management.

### The surrogate with the most similar cross-contamination dynamics to *Salmonella* varied depending on the scenario

Microbial surrogates have traditionally been used to estimate the lethality of various process controls on relevant foodborne pathogens. In this framework, a good surrogate is characterized by having similar, or slightly more conservative, resistance characteristics to the mode of inactivation in the pathogen of interest under a given control (72). Additionally, the surrogate should have similar variability in response to the mode of inactivation (72). *Salmonella* surrogates, such as *E. faecium* NRRL B-2354 (73), have been used to validate thermal processes based on their resistance to thermal inactivation. However, for dry cleaning, the relevant characteristic is transfer and removal during wiping, brushing, scraping, or flushing of surfaces. We used modeling outputs as an exploratory framework for surrogate evaluation in dry cleaning.

**Fig 7:**
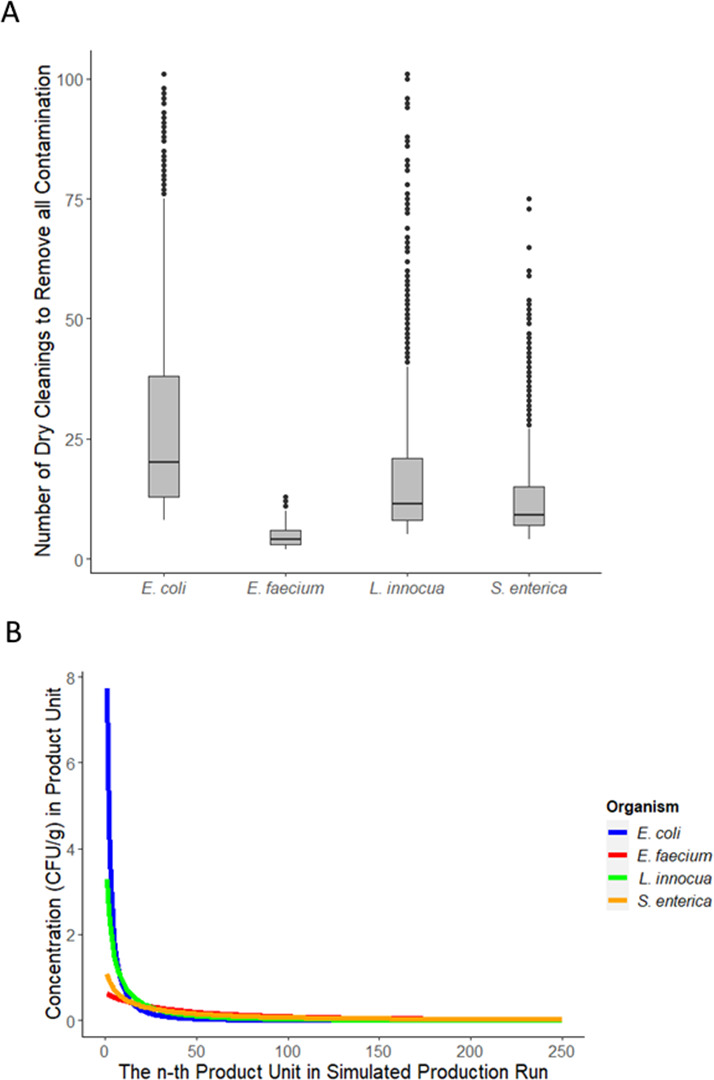
(A) Each data point (*n* = 1,000 iterations) represents the number of successive cleanings with a dry towel required to remove all surface contamination (4 log) as modeled in scenario 4. The number of cleanings required was the most similar between *L. innocua* and *S. enterica*. (B) Across 1,000 simulated production runs (scenario 1, 2-log CFU), the median contamination concentration (CFU/g) in the n-th sequential milk powder unit produced during a production run is graphed for each surrogate. *S. enterica* and *E. faecium* had the most similar diffusion of contamination into product, with less contamination being transferred into each unit, but more units becoming contaminated*.*

Experimental transfer data were generated for the following three potential *Salmonella* surrogates: *E. coli* ATCC 25922, *E. faecium* NRRL B-2354, and *L. innocua* ATCC 51742 (File S1) and used to re-run the simulations described in scenarios 1, 2, and 5 (Table 1). Scenario 5 simulated the repeated dry wiping of a contaminated surface until all surface contamination (4-log CFU) was removed and assumed no contamination was re-introduced (Fig. 7A). Across simulations, the median number of dry towel cleanings (three passes of the towel per cleaning) required to remove all *Salmonella* was 9 [5, 35]. The most similar surrogate was *L. innocua* which took a median of 12 [6, 64] dry towel cleanings to remove all the surface contamination. *E. faecium* took only a median of 4 [3, 9] dry towel cleanings to remove all contamination, which means using it as a surrogate in dry towel cleaning studies may overestimate *Salmonella* reduction. On the other hand, a median of 20 [10, 101] dry towel cleanings were required to remove all *E. coli* contamination. Therefore, *E. coli* would be a very conservative surrogate for the removal of *Salmonella* for this method of dry cleaning. The variability in the outcomes should also be considered. Although *L. innocua* was the most similar to *Salmonella* in terms of the median number of cleanings to remove all contamination, the variability between simulations was higher (standard deviation: 20 for *L. innocua* and 10 for *Salmonella*). This difference in variability introduces uncertainty in the pathogen-surrogate relationship that should be accounted for when data from surrogates is used to validate dry cleaning.

**Fig 8:**
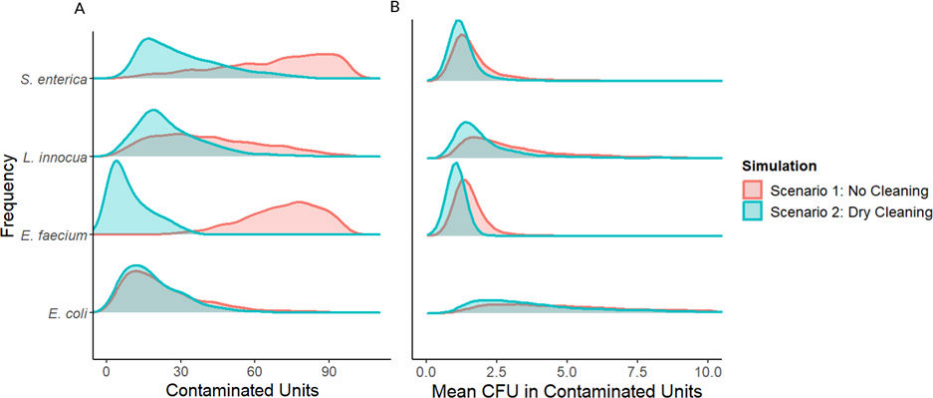
Density estimate of the prevalence of contaminated milk powder units (A) and the mean concentration within contaminated units (B) across all simulated production runs (*n* = 1,000 iterations) for each organism after a 2-log CFU contamination breach. Higher peaks mean more simulations fell into the range measured on the x-axis. In terms of contaminated units, *Salmonella* and *E. faecium* were the most similar in the absence of a towel-wiping intervention, but *L. innocua* was more similar to *Salmonella* when towel wiping was included*.*

The microbial log reduction (*M_r_*) from dry wiping is the only input parameter in scenario 4; therefore, these results reflect the difference in measured log reductions of each bacterium after dry towel cleaning. For example, *E. coli* had the lowest average log reduction (Table 1), which corresponds to the greatest number of dry towel cleanings required to remove all surface contamination. By contrast, in scenario 1 (no dry cleaning intervention), the transfer coefficient from the contaminated surface into milk powder (*T_c_*) is the only parameter that is variable between surrogates. In this case, *E. faecium* behaved most like *Salmonella* (Fig. 7B). Both showed a pattern of slow transfer from the surface into milk powder, resulting in a greater number of contaminated units with less contamination in those units on average than *L. innocua* and *E. coli*. The number of contaminated units across all scenario 1 simulations for a 2-log CFU contamination breach was 72 [24, 96] for *Salmonella*, 19 [5, 56] for *E. coli*, 74 [44, 93] for *E. faecium*, and 39 [11, 80] for *L. innocua* (Fig. 8A). By this metric, the results from *E. faecium* had a similar number of cross-contaminated milk powder units as *Salmonella* and similar variability among simulated production runs. This is in line with the measured transfer rates from the stainless-steel surface to milk powder, which were higher for *E. coli* and *L. innocua* (Table 1). Similarly, the average concentration of *Salmonella* in contaminated units was −2.33-log CFU/g [-2.46, –1.86], which was the most similar to *E. faecium* −2.35-log CFU/g [-2.45, –2.12]. In scenario 2 (flushing intervention followed by production), the transfer coefficient from the contaminated surface into milk powder (*T_c_*) is again the only model parameter that is variable between surrogates. Therefore, like scenario 1, contamination outcomes were most similar between *Salmonella* and *E. faecium*. After a 2-log CFU contamination breach followed by flushing with 150 kg of milk powder, the number of units contaminated with *Salmonella* was 0 [0, 41]. For *E. faecium,* this value was 0 [0, 19]. For *L. innocua* and *E. coli*, the number of contaminated units was 0 [0, 1] and 0 [0, 0], respectively.

In scenario 3 simulations (dry wiping intervention followed by production), both the dry towel microbial reduction (*M_r_*) and surface to milk powder transfer (*T_c_*) are used as model parameters. In this scenario, both *L. innocua* and *E. faecium* were similar to *Salmonella* depending on the outcome of interest. Simulations with *Salmonella* input data resulted in 27 [12, 64] contaminated units while using *L. innocua* data resulted in 22 [8, 53], the most similar in terms of the number of contaminated units (Fig. 8A). *E. faecium* and *E. coli* simulations resulted in 7 [1, 26] and 16 [4, 42] contaminated units, respectively, in scenario 3. In terms of the contamination concentration within units, *L. innocua* and *E. faecium* were similar to *Salmonella*. The average contamination concentration in contaminated units was −2.43-log CFU/g [–2.48, –2.08] for *Salmonella* in scenario 2. For *E. faecium*, *L. innocua,* and *E. coli,* these values were –2.48 [–2.48, –2.38], –2.25 [–2.45, –1.72], and –1.89 [–2.32, –1.28] log CFU/g, respectively.

We found that the surrogate most representative of *Salmonella* depended on the scenario being modeled and changed as multiple model parameters were considered. For example, in scenario 4 where only dry wiping was considered, the degree of dry wiping needed to remove all contamination from a surface that was most similar to *Salmonella* was *L. innocua* (Fig. 7A), although differences in variability could complicate the pathogen-surrogate relationship. In scenario 1, which simply evaluated transfer into product, *E. faecium’*s results were the most similar to *Salmonella* and had similar variability (Fig. 8A and B). When a dry wiping intervention was applied before production (scenario 3), *L. innocua* resulted in the most similar number of contaminated product units and similar variability across simulations. In summary, *L. innocua* performed better as a surrogate for dry towel wiping, but *E. faecium* performed better as a surrogate when modeling surface transfer to milk powder during production. For a production run preceded by a dry towel wiping intervention (scenario 3), the most similar surrogate in terms of the number of contaminated units was again *L. innocua*, although *E. faecium* was also similar in terms of contamination within units. Therefore, the most appropriate surrogate changed based on the exact scenario being modeled, the associated parameters for that scenario, and the outcome of interest. Notably, these surrogate comparisons do not accurately reflect all possible dry cleaning applications. Other relevant parameters that should be explored include surface material type, the material used for flushing, removal from other cleaning tools such as brushes and scrapers, and transfer from different types of niches. Therefore, the surrogate evaluation presented here begins to explore the dynamics relevant to a surrogate for dry cleaning, but many other variables remain to be studied in future work.

Direct comparison of empirical transfer and reduction data is often challenging because it is unclear how small differences in these values will manifest in real-world cleaning outcomes (72). By contrast, model outputs are more interpretable to industry or regulators and are regularly used as decision support tools. Comparing the predicted number of contaminated units and the concentration of contamination within those units before and after cleaning for different surrogates has a clearer connection to the outcome of interest than comparison among, for example, the means of transfer coefficients. For example, QMRA studies could consider how using transfer data from different *Salmonella* surrogates would affect downstream modeling outcomes like the number of contaminated servings in products and human exposure. Similarly, how the variability of microbial reduction during dry wiping manifests as a different range of potential outcomes gives context for assessing different surrogates for use in dry cleaning validation experiments.

In-plant validation data will be needed to fully realize the advantages offered through simulation modeling. This is a crucial role for the use of surrogates which may be introduced into commercial and pilot-scale systems. Under some circumstances, for example, treatments using milk powder flushing, *E. faecium* may be a suitable candidate in place of *Salmonella*. On the other hand, an experimental study evaluating the efficacy of dry wiping (using the towel material evaluated here, at least) for *Salmonella* removal should not be studied using *E. faecium* as a surrogate because the results will overestimate the efficacy of the intervention against *Salmonella*. Additionally, the choice of non-pathogenic surrogate should also be weighted on the impact of environmental monitoring programs, since *E. faecium* (lactic acid bacteria) and *L. innocua* (*Listeria* spp.) may be included among hygienic indicators in an environmental monitoring program.

### Conclusion

Overall, these results highlight the utility of modeling for assessing dry cleaning strategies and selecting appropriate surrogates for *Salmonella* during dry transfer and removal. Both flushing and, to a lesser degree, wiping with a dry towel reduced the prevalence and concentration of *Salmonella* in milk powder units following contamination. A limitation of the current model is its use of smooth, flat surfaces as the point of environmental contamination. Future research should explore the impact of niches and dead zones by measuring their unique cross-contamination dynamics. Sensitivity analysis underscored the influence of both the surface-to-food material transfer coefficient and the proportion of food material that contacts the contaminated surface on model outcomes. Therefore, accurate data on these parameters in more complex niches will be essential for understanding flushing and cross-contamination in the processing line more broadly. Future modeling applications may also consider the trade-off between enhanced microbial reduction and potential microbial growth that occurs during intermittent wet sanitation and how these risks compare based on the frequency of wet sanitation breaks. Our results indicated that the most appropriate surrogate for *Salmonella* dry transfer depended on the specific transfer scenario, reinforcing that there is no one-size-fits-all surrogate. Robust validation of the model through future studies, including the use of surrogate organisms, is vital for ensuring the accuracy of the model in real-world applications.

## AFTER EPUB

*[This article was published on 17 April 2025 without Acknowledgments. The Acknowledgments were added in the current version, posted on 21 May 2025.]*

## SUPPLEMENTAL MATERIAL

Supplemental material aem.02086-24-s0001.xlsx Microbial transfer and reduction data used in simulation modeling.

ASM does not own the copyrights to Supplemental Material that may be linked to, or accessed through, an article. The authors have granted ASM a non-exclusive, world-wide license to publish the Supplemental Material files. Please contact the corresponding author directly for reuse.
